# Re-sleeve gastrectomy is a safe and sensible intervention in selected patients: retrospective cohort study

**DOI:** 10.1097/JS9.0000000000000743

**Published:** 2023-09-13

**Authors:** Lionel El Khoury, Jean-Marc Catheline, Malak Taher, Joel Roussel, Yasmina Bendacha, Rodolfo Romero, Rami Dbouk, Regis Cohen

**Affiliations:** Department of Digestive Surgery, Centre Hospitalier de Saint-Denis, 2 rue du Docteur Delafontaine, Saint-Denis, France

**Keywords:** bariatric surgery, obesity, re-sleeve gastrectomy, revised sleeve gastrectomy, revisional surgery

## Abstract

**Introduction:**

Sleeve gastrectomy is a good treatment intervention to control the metabolic syndrome in patients with obesity worldwide. However, weight regain is of great concern and would usually necessitate a reintervention. In recent years, re-sleeve gastrectomy (ReSG) has been proposed to treat weight regain in the context of a large residual stomach. Our objective was to analyze the long-term results and safety profile of this intervention in a large case series.

**Methods:**

From September 2010 to March 2021, a retrospective cohort study in a tertiary nonuniversity hospital was performed. Seventy-nine patients received a ReSG by laparoscopy. Preoperative radiologic imaging showed a dilation of the gastric pouch exceeding 250 cc in all cases.

**Results:**

A total of 79 patients (87% females) with a mean age of 44.8 years old and a mean BMI of 40.0 kg/m^2^ were enrolled in the study. The mean follow-up was 44.8 months. The ReSG indication was insufficient weight loss in 37 patients (46.8%) and weight regain in 39 patients (53.2%). The authors noticed a 10.1% complications rate: gastric stenosis (5.1%), bleeding (2.5%), and incisional site hernia in 2.5%, with no death. There was no gastric fistula detected. The mean BMI decreased to 33.1 kg/m^2^ after ReSG (a decrease of 6.9 kg/m^2^).

**Conclusion:**

After insufficient weight loss or weight regain following sleeve gastrectomy and in the presence of localized or global gastric tube dilation, ReSG seems to be a good treatment choice and a safe procedure.

## Introduction

Highlights

- There is no consensus on the best treatment option after sleeve gastrectomy failure.
- We report our experience of re-sleeve gastrectomy (ReSG) in 79 selected patients (44.8 months follow-up).
- The mean BMI decreased to 33.1 kg/m^2^ after ReSG (a decrease of 6.9 kg/m^2^) with no leaks and no death.
- ReSG seems to be a good treatment choice and a safe procedure in selected patients.

Currently, sleeve gastrectomy (SG) has proven its efficiency in weight loss, the decrease of obesity-related complications, and survival. It has become extremely popular worldwide due to its easier technical approach and safety profile compared to other procedures^1–3^. Despite the wide expansion of bariatric surgery and SG, weight regain or insufficient weight loss after surgery is a matter of concern, usually requiring revisional surgery in 2.4 to 19.9% of patients^4^. According to the literature, the preferred interventions after SG are Roux-en-Y gastric bypass (RYGB or one-anastomosis gastric bypass (OAGB)^5^, biliopancreatic diversion with duodenal switch^6^, or the newly developed single anastomosis duodeno-ileal bypass^7^ (SADI) technique. There is currently no consensus on the best treatment option after bariatric surgery failure, or which patients may benefit from revisional surgery despite the high risks involved^8^. In 2003, Gagner and Rogula^9^ was the first to suggest re-sleeve gastrectomy (ReSG) for insufficient weight loss after biliopancreatic diversion with duodenal switch. Baltasar *et al*.^10^ in 2006 concluded that ReSG could be possible after gastric tube dilation or insufficient initial sleeve volume reduction. Subsequent reports have shown the efficacy and safety of ReSG in achieving short-term weight loss^11–14^. The aim of our study was to present our experience of ReSG, to assess its efficacy, and assess whether ReSG should be a preferred treatment option for patients meeting specific criteria and presenting weight-loss failure or weight regain after initial SG, with an anatomically larger stomach.

## Methods

### Patients

From September 2010 to March 2021, a retrospective cohort study in a tertiary nonuniversity hospital was performed. It was conducted in accordance with the Good Clinical Practice guidelines, the principles of the Declaration of Helsinki, and to the strengthening the reporting of cohort, cross-sectional and case-control studies in surgery (STROCSS) 2019 Guideline^15^. Ethical approval for this study (Ethical Committee N° 0040_CHIRURGIE BARIATRIQUE_RESLEEVE) was provided by the Ethical Committee of Saint-Denis Hospital, France on 16 March 2023. Written informed consent was obtained from the patient for publication and any accompanying images. A copy of the written consent is available for review by the Editor-in-Chief of this journal on request.

ReSG was decided after multidisciplinary assessment and preparation involving bariatric surgeons, nutritionists, dietitians, and psychologists. As a reminder, insufficient weight loss was defined as a loss of under 50% of excess weight loss (EWL) 18 months after surgery^16^ and weight regain was defined as gradual weight regain after initial weight loss in excess of 50% of EWL^16^. ReSG was considered for patients with insufficient weight loss or weight regain after the initial sleeve, only if they did not have severe reflux symptoms or severe esophagitis and presented a dilated gastric pouch (as evidenced by radiological findings). Reflux symptomatology was evaluated through a questionnaire, and the effectiveness of proton-pump inhibitors was noted. All patients underwent a preoperative upper gastric endoscopy, and none had a pH manometry.

**Figure 1:**
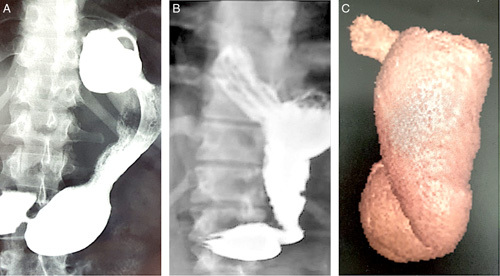
After barium swallow contrast study (A, B) and computed tomography scan volumetric (C). The gastric dilation was classified as primary localized (A, B) or secondary global dilation (C).

To evaluate post-SG stomach volume and anatomy, all patients underwent a barium swallow contrast study. After 2014, 58 patients (73.4%) also underwent volumetric computed tomography scans (gastroscanner). ReSG was suggested when the gastric volume exceeded 250 cc on the gastroscanner, as proposed by a French team in 2014^16^. For the volumetric computed tomography scans, patients ingested an effervescent powder mixture to distend the stomach, and images were acquired at 30 and 60 s. Volumetric reconstructions of the gastric cavity were performed using Myrian software. Gastric volume measurements were obtained either automatically or manually. The gastric dilation was classified as primary localized dilation of an improperly dissected fundus region during initial sleeve (Fig. 1A, Fig. 1B) or secondary global dilation defined by a global homogeneous gastric dilation due to insufficient initial sleeve calibration or a secondary natural dilation after surgery (Fig. 1C)^17^.

### Surgical procedure

All cases were operated via laparoscopy by a single surgeon (J.M.C.) with no conversion to laparotomy. Multiple ports were used to facilitate exposure. The left liver lobe was dissected carefully from the anterior surface of the stomach toward the hiatus, and the previous staple line on the greater curvature was exposed entirely until the left crus became visible. The dissection was performed using a Ligasure sealing device (Valleylab). A calibration tube of 34 Fr was inserted and the stapling was done via endo-GIA (Medtronic) either in SG or ReSG, with the use of a tri-staple device after 2012 (black 32.9% or purple 48.1%), and a green cartridge were used (19%) before 2012. The methylene blue leak test was performed as a routine. Reinforcement of the staple line was achieved in 84.8% of cases and performed through a continuous running suture (65.8%) or by Seamguard (W.L. Gore & Associates) in 19% of the last cases. We preferred considering abdominal drainage after revisional surgery and 96.2% underwent drainage for up to three days after surgery.

### Theory/Calculation

Our theory was that patients undergoing ReSG revision surgery had good weight outcomes and few complications. We present our retrospective experience.

## Results

Between September 2010 and March 2021, a total of 2252 SG procedures were performed at our institution, including 2173 initial SG and 79 ReSG cases, accounting for 3.5% of all SG procedures. Among the 79 patients who underwent ReSG, there were 69 women and 10 men, with a mean age of 44.8 years (ranging from 23 to 64 years) and a mean BMI of 40.0 +/− 5.2 kg/m^2^ (ranging from 34.1 to 64.5 kg/m^2^). The mean gastric volume measured in our patient series was 412.2 cc (ranging from 255 to 1050 cc).

Out of the 79 patients, 32 (40.5%) had a history of gastric band placement, while the rest were naïve to any prior bariatric surgery. Among them, 26 patients (32.9%) had their initial SG performed at another hospital. The mean operative time for ReSG was 118 min (ranging from 70 to 188 min), and the mean hospital stay was 5.1 days (ranging from 3 to 20 days).

Complications were observed in eight cases (10.1%) following ReSG. Gastric stenosis was the most common complication, occurring in four cases (5.1%), with successful treatments including endoscopic dilation and RYGB. Other complications included bleeding in two cases (2.5%), both of which were managed laparoscopically, and incisional site hernia in two cases (2.5%), which required surgical intervention. Notably, there were no cases of staple line leakage or mortality in our study.

**Figure 2:**
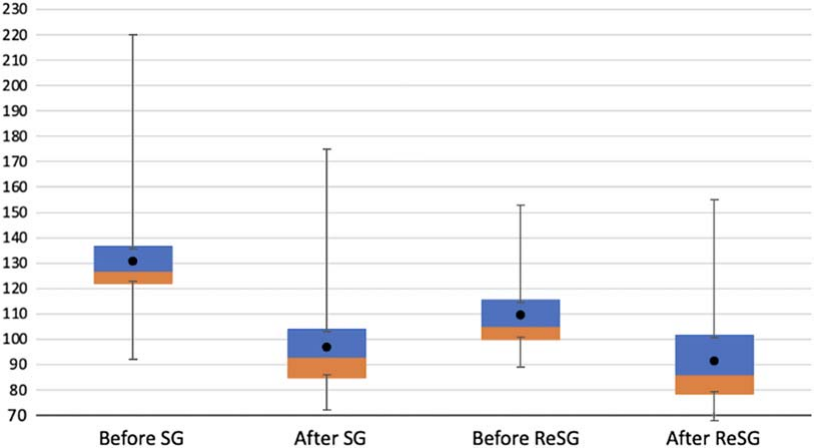
Mean weight before/after SG, and before/after ReSG. ReSG, re-sleeve gastrectomy; SG, sleeve gastrectomy.

**Figure 3:**
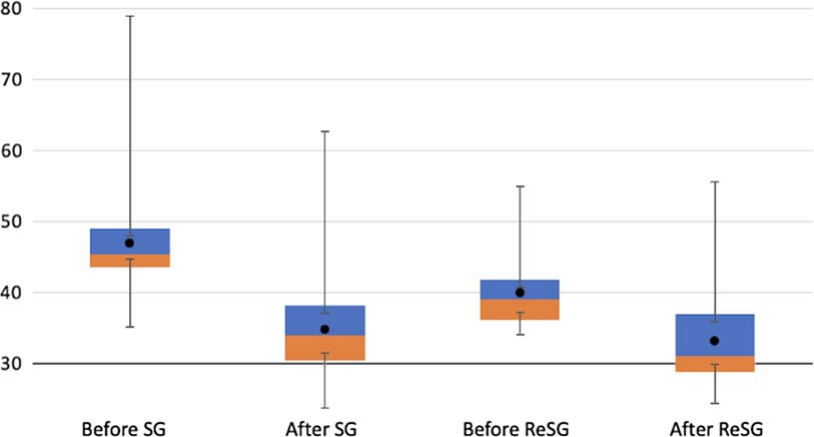
Mean BMI before/after SG, and before/after ReSG. ReSG, re-sleeve gastrectomy; SG, sleeve gastrectomy.

The maximal weight loss after the initial sleeve was achieved at 19.8 months (mean time when the lowest weight was observed, range: 14–27) and the mean time between SG and ReSG was 37.4 month (range:18–80). The mean follows up after ReSG was 44.8 months (mean time between ReSG and last consultation, range 8–126). The mean weight before ReSG was 109.5 +/− 15.4 kg (range 89–180 kg) and became 91.2+/−16.4 kg (range: 68–155) after a mean follow-up of 44.8 months (median 45 months) after ReSG. The mean BMI was 40.0 +/−5.2 kg/m^2^ (range 34.1–64.5) before ReSG and decrease to 33.1+/−5.9 kg/m^2^ (range 24.4–55.6) after ReSG. The mean percentage of EWL after initial SG was 56.9+/−12.7% (range: 27.3–73) and of 47.9+/−2.9% (range 33.1–64.1) after ReSG (Table 1). At 7 years following the initial SG, the mean weight loss decreased from 130.8 to 91.2 kg after the ReSG (Fig. 2), and the mean BMI decreased from 47 to 33.1 kg/m^2^ (Fig. 3) (Table 1). There was no correlation between the fact that it was a primary or secondary dilation. The type of dilation was not a predictive criterion of the result after ReSG. There was no correlation between preoperative pouch volume and weight loss after ReSG. Nine patients had reflux symptoms before (11.4%) and 20 after ReSG (25.3%). After ReSG, patients had usually mild reflux, controlled by PPIs. None had severe esophagitis nor reflux symptoms calling for RYGB.

**Table 1:** Weight, BMI, and %EWL before and after each procedure.

	Before SG^a^	After SG^b^	Before ReSG^c^	After ReSG^d^
Mean weight (Kg) Extreme range	130.8+/−17.6 92–220	96.8+/−15.6 72–175	109.5+/−15.4 89–180	91.2+/−16.4 68–155
Mean BMI (Kg/m^2^) Extreme range	47.0+/−6.2 35.1–78.9	34.7+/−5.7 23.8–62.7	40.0+/−5.2 34.1–64.5	33.1+/−5.9 24.4–55.6
Mean %EWL Extreme range		56.9+/−12.7 27.3–73.0		47.9+/−2.9 33.1–64.1

a One month before SG.

b 19.8 months after SG.

c 37.4 months after SG.

d 82.2 months after SG and 44.8 months after ReSG.

SG, sleeve gastrectomy; ReSG, re-sleeve gastrectomy; EWL, excess weight loss.

## Discussion

Our study involving a large series of patients with primary gastric dilation who underwent long-term follow-up demonstrated significant weight loss after ReSG (BMI decreased from 40.0 before ReSG to 33.1 kg/m^2^ after ReSG). The number of complications was low (10.1%) with no death, compared to other studies of ReSG described in a large systemic review and meta-analysis^18^.

Our weight loss results were similar although durable with a mean follow-up of 44.8 months. Moreover, we had zero staple line leakage, which could be due to the large experience, the learning curve or even the evolution of the technique and surgical material. Obesity is a chronic disease with a very complicated pathophysiology which necessitates a multidisciplinary approach including psychological and eating behavior evaluation. ReSG is supposed to intervene on the anatomical part of the problem to decrease stomach residual volume in order to offer a satiety sensation in association of the decreased ghrelin levels (orexigen hormone) by removing totally the gastric fundus. There is probably many other unknown mechanisms^19^. Even if SG is a well standardized surgical technique nowadays, it is very easy to do it incorrectly and in an improper way. The cause of stomach dilation after initial SG is not well understood and defined^20^. Primary gastric dilation is referred to the presence of a large gastric fundus whereas secondary dilation is a global entire stomach enlargement. The mechanisms of primary dilation could be explained by an improper posterior dissection during initial SG and an improper total fundal removal due to the inexperience of the surgeon and/or the difficulty in some cases of superobese male patient or with a history of a previous gastric band or maybe due to a neofundus formation. Moreover, global enlargement could be due to improper initial tube calibration or in relation with the natural evolution of the stomach after SG^20,21^. Weight regains after SG are common; however their etiologies remain poorly understood including genetic, dietary, natural history, and psychiatric factors^19^. After SG, some studies related directly and positively the residual gastric volume to weight regain^19,20^. The barium swallow contrast studies along with computed tomography scanner volumetry are of great interest to evaluate properly the anatomy of the remnant stomach. We and other colleagues^17^ consider large a gastric volume exceeding 250 cc, cut off to reconsider ReSG. Moreover, Al-Sabah *et al.* ^22^ consider the ability to perform easily a retroflex during upper endoscopy a sign of gastric remnant dilation considering ReSG. In an interesting paper, Braghetto *et al*.^23^ showed that mean gastric volume increases 36 months after initial SG (from 108 to 250 cc) and that none of the 15 patients’ experiences weight regain whereas Langer *et al*.^24^ have noted many weight regainers without evidence of stomach dilation at a mean follow-up of 20 months. As others, we are convinced that patients eating habits and behavior along with psychological assessment are of importance^25^. The multidisciplinary evaluation before redo-surgery is the cornerstone of a good indication. Only a large randomized study with revisional surgery versus nonsurgical support could demonstrate the influence of ReSG in good results.

**Figure 4:**
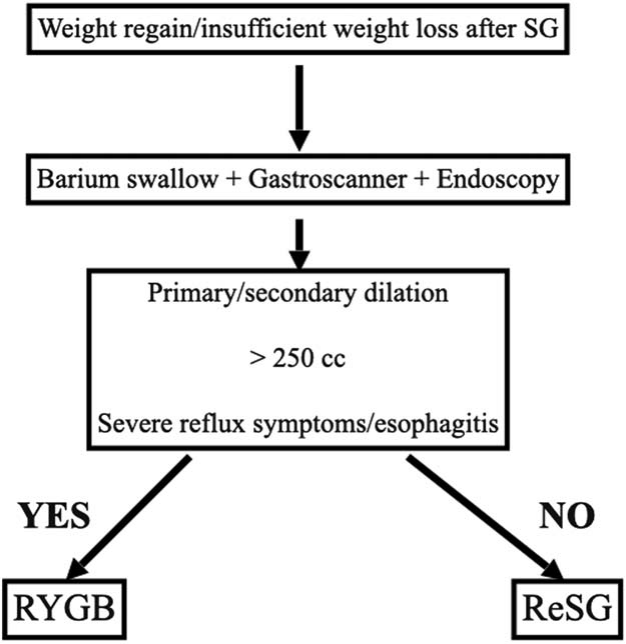
Our institutional algorithm for revisional surgery after SG. SG, sleeve gastrectomy.

If bariatric surgery is gaining progress worldwide and that the number of procedures is increasing, revisional surgery is increasing in parallel and is found to be around 2.4 to 19.9% after SG^4^. The proper redo-procedure to select is not standardized and need to be defined; however, in our specialized metabolic unit with experienced bariatric surgeons we propose the algorithm shown in Figure 4 where we consider ReSG in patients presenting with localized/generalized gastric dilation along with >250 cc and without significant reflux or esophagitis. Many authors wonder about the efficiency of performing a restrictive surgery for patients who have already undergone a bariatric restrictive procedure. What if the initial restriction was not very restrictive? Or what about secondary insufficient restriction? Does not this deserve reconsideration another chance with restrictive surgery before passing step to a more complex malabsorptive bariatric procedure?

The gold standard of revisional surgery remains RYGB after SG^26^. In a recent systematic review and metanalysis^3^, revisional strategies for weight regain or insufficient weight loss following SG were compared. All procedures have been found to have a relevant weight loss during the follow-up. Between ReSG, endoscopic gastroplasty, RYGB, OAGB, SADI, and duodenal switch, the best results in terms of complications rate and efficacy were in favor of OAGB which is currently prohibited in France. In these studies that should be interpreted with caution due to the number of patients and the methodologies, ReSG seemed to have the efficiency of redo RYGB with much less complications^3^.

Beside the effectiveness of the ReSG, we must point out the low level of complications. Gastric stenosis after ReSG could be due to the stapling of gastric tissue under tension, in front of the angulus narrowing so much the stomach along the calibration tube in that specific area. Nocca *et al.* ^27^ noted 12% of gastric stricture following ReSG and proposed that stapling should be done on the antrum then in the upper part of the stomach starting above the incisura angularis leaving that area without stapling it. However, surgical skills are highly recommended to perform this revisional surgery. Compared with malabsorptive procedures, ReSG offers a chance to the patient to restore a « proper » sleeve and presents the advantage of having no or less dumping syndrome along with all the advantages of restrictive procedures^28–30^ especially with no lifelong vitamin intake. It should be planned according to patient preference and the surgeon’s experience since there is no ʻone size fits allʼ as a miraculous solution for obese patients who fail their first attempt SG. Many studies^31–33^ regarding laparoscopic ReSG have been published in the literature and tend to demonstrate the good results of ReSG in the short and midterm follow-up with an equivalent overall complications rate comparable to that after SG. In terms of weight loss, Al-Sabah *et al*.^22^ compared ReSG to gastric bypass as a revisional surgery after laparoscopic initial SG and showed better results of ReSG on a short-term follow-up of 1 year. Other comparative study at 1 year demonstrated a similar BMI loss (kg/m^2^) of 4.7 in ReSG vs 4.6 in RYGB, with a rate of complications at 90 days of 0 versus 14.6%^13^. The results of these studies are presented in Table 2. These results should be interpreted with caution. While our study, represents the longest follow-up of ReSG as revisional surgery after SG, larger prospective and comparative studies with longer follow-up are needed to demonstrate the relevance of each revisional procedures^3^. Limitations of the study include its observational retrospective design and its one center reference.

**Table 2:** Characteristics of patients after ReSG according to a systematic review^16^.

	Year	Patients number (*n*)	BMI before ResG (kg/m^2^)	BMI after ResG (kg/m^2^)	Leak (*n*)	Follow-up (months)
Ianelli *et al*.^11^	2011	13	34.9	27.5	0	12
Cesana *et al*.^34^	2014	11	38.9	32.2	0	12
El Ansari W.^16^	2015	61	39	29.8	0	19.9
Sileccia *et al*.^21^	2015	19	36.5	28.8	2	24
Nett *et al*.^35^	2016	17	39.8	33.8	1	37.2
Rebibo *et al*.^36^	2018	46	41.2	32.1	5	22
De Angelis *et al*.^14^	2018	19	44.3	27.8	NA	52
Antonopoulos *et al*.^37^	2019	61	40.5	31.6	5	12
Mehmet^38^	2019	21	46.1	24.5	0	12
Filip *et al*.^39^	2019	27	35.7	27.65	0	36
Al-Sabah *et al*.^22^	2020	24	42	34	0	12
Bonaldi *et al*.^40^	2023	102	38.6	30.4	3	55
Our study	2023	79	40	33.1	0	44.8
Total (sum/mean)		500	40.3	34	16	32

NA, not available; ReSG, re-sleeve gastrectomy.

## Conclusion

ReSG seems to be a good procedure to be included in the panel of choice of revision surgery because of its safety and good results on total weight loss in selected patients. More studies must compare revisional surgery available with a greater number of patients to implement evidence-based guidelines. To date, prospective clinical trials adequately done to determine the most effective treatment in the setting of inadequate weight loss after SG are lacking.

## Ethical approval

Ethical approval for this study (Ethical Committee N° 0040_CHIRURGIE BARIATRIQUE_RESLEEVE) was provided by the Ethical Committee of Saint-Denis Hospitals, France on 16 March 2023.

## Consent

Written informed consent was obtained from the patient for publication and any accompanying images. A copy of the written consent is available for review by the Editor-in-Chief of this journal on request.

## Sources of funding

No funding.

## Author contribution

L.E.L., J.M.C., M.T., J.R., Y.B., R.R., R.D., and R.C.: contributed to the data collection, reviewed and edited the manuscript, and approved its submission; L.E.L., J.M.C., and R.C.: wrote the first draft of the manuscript; J.M.C.: designed the study; L.E.L., J.M.C., M.T., Y.B., R.R., and R.D.: contributed to the surgery procedures; J.M.C.: designed and performed the statistical analyses; L.E.L., J.M.C., and R.C.: co-supervised the study; L.E.L., J.M.C., and R.C.: are the guarantors of this work and, as such, had full access to all the data in the study and take responsibility for the integrity of the data and the accuracy of the data analysis.

## Conflicts of interest disclosure

No potential conflicts of interest relevant to this article were reported.

## Research registration unique identifying number (UIN)

researchregistry9102. https://researchregistry.knack.com/researchregistry#home/registrationdetails/6478283087a4bd0026e11d4e/.

## Guarantor

Regis Cohen. E-mail: cohen.regis93@gmail.com.

## Data availability statement

Data Statement and XLS file are available.

## Provenance and peer review

We confirmed that the paper was not invited If published you can write: ʻNot commissioned, externally peer-reviewed.
